# Directional Electromagnetic Interference Shielding Based on Step-Wise Asymmetric Conductive Networks

**DOI:** 10.1007/s40820-021-00743-y

**Published:** 2021-12-06

**Authors:** Bai Xue, Yi Li, Ziling Cheng, Shengdu Yang, Lan Xie, Shuhao Qin, Qiang Zheng

**Affiliations:** 1 Department of Polymer Materials and Engineering, College of Materials and Metallurgy, Guizhou University, Guiyang, 550025 People’s Republic of China; 2 National Engineering Research Center for Compounding and Modification of Polymer Materials, National and Local Joint Engineering Research Center for Functional Polymer Membrane Materials and Membrane Processes, Guiyang, 550014 People’s Republic of China; 3 College of Polymer Science and Engineering, Zhejiang University, Hangzhou, 310027 People’s Republic of China; 4 College of Materials Science and Engineering, Taiyuan University of Technology, Taiyuan, 030024 People’s Republic of China

**Keywords:** Solution encapsulation, Step-wise asymmetry, Directional electromagnetic interference shielding, Electrical conductivity, Vacuum-assisted self-assembly

## Abstract

Some precision electronics such as signal transmitters need to not only emit effective signal but also be protected from the external electromagnetic (EM) waves. Thus, directional electromagnetic interference (EMI) shielding materials (i.e., when the EM wave is incident from different sides of the sample, the EMI shielding effectiveness (SE) is rather different) are strongly required; unfortunately, no comprehensive literature report is available on this research field. Herein, Ni-coated melamine foams (Ni@MF) were obtained by a facile electroless plating process, and multiwalled carbon nanotube (CNT) papers were prepared via a simple vacuum-assisted self-assembly approach. Then, step-wise asymmetric poly(butylene adipate-co-terephthalate) (PBAT) composites consisting of loose Ni@MF layer and compact CNT layer were successfully fabricated via a facile solution encapsulation approach. The step-wise asymmetric structures and electrical conductivity endow the Ni@MF/CNT/PBAT composites with unprecedented directional EMI shielding performances. When the EM wave is incident from Ni@MF layer or CNT layer, Ni@MF-5/CNT-75/PBAT exhibits the total EMI SE (SE_T_) of 38.3 and 29.5 dB, respectively, which illustrates the ΔSE_T_ of 8.8 dB. This work opens a new research window for directional EMI shielding composites with step-wise asymmetric structures, which has promising applications in portable electronics and next-generation communication technologies.

**Supplementary Information:**

The online version contains supplementary material available at 10.1007/s40820-021-00743-y.

## Highlights

- Ni@MF/CNT/PBAT composites with step-wise asymmetric structures are fabricated via a facile solution encapsulation approach.
- The composites exhibit the unprecedented directional electromagnetic interference shielding performances (ΔSE_T_ = 8.8 dB), which are further verified by an actual application measurement in a remote controlled toy car system.

**Supplementary Information:**

The online version contains supplementary material available at 10.1007/s40820-021-00743-y.

## Graphical abstract

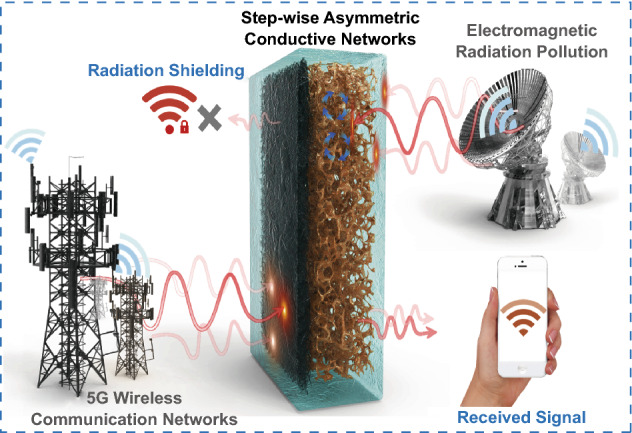

## Introduction

Given the prosperity of electronic devices and modern telecommunication technology that are represented by newly emerged 5G wireless system, electromagnetic interference (EMI) pollution has become an inevitable and serious social issue that has greatly detrimental effects both on the operation of precision instruments and the health of human beings [1–4]. In order to attenuate undesirable electromagnetic (EM) wave, it is imperative and urgent to exploit high-performance EMI shielding materials [5–8]. Traditionally, metal-based materials are widely used in efficient EMI shielding because of the high conductivity and/or permeability, but intrinsic high density, easy corrosion, non-flexibility, and difficult processing make them less desirable for smart and subtle electronics [9–11]. As a consequence, it has created a crucial demand for developing new lightweight flexible EMI shielding materials with favorable mechanical properties.

In recent years, conductive polymer composites (CPCs) comprising polymer matrix and conductive fillers have drawn a wide of research interest and emerged as promising candidates in next-generation protection devices in terms of their superiorities such as light weight, low cost, flexibility, easy processing, and corrosion resistance [12–15]. Gu et al. [16] fabricated multifunctional flexible EMI shielding silver nanowires (AgNWs)/cellulose films via vacuum filtration and hot-pressing process. The AgNWs/cellulose film with 44.5 μm exhibited superior EMI shielding effectiveness (SE) of 101 dB, excellent thermal conductivity of 10.55 W mK^−1^, and outstanding Joule heating performances at a 50 wt% loading fraction. As is well known, EMI SE of CPC materials is highly dependent on the electrical conductivity that could induce the enhancement of impedance mismatch and dielectric loss for EM wave. Thus, high filler content and/or sample thinness are fundamental to acquire ideal EMI SE of CPCs, leading to the reduction of flexibility, mechanical performances, and processability [17–19]. So, it remains a difficult task to develop high-efficient polymer-based EMI shielding materials with wonderful flexibility and mechanical properties.

As the structure of a material always determines its properties, structural design and controlling without sacrificing other merits are of great significance for realizing high-performance EMI shielding [20–24]. Recent researches have verified that various types of structures that are beneficial to EMI shielding are successfully constructed [25–29]. Therein, segregated structure is able to concentrate conductive fillers together to build conductive networks in polymer matrix, which accordingly reduces the conductive percolation threshold [30–34]. Li et al. [35] designed the segregated structure composed of magnetic hybrid (rGO@Fe_3_O_4_) cores and CNT walls, which reached an EMI SE of 43.5 dB and SE_A_/SE_T_ ratio of ~ 90% at 4 wt% CNT/5 wt% rGO@Fe_3_O_4_ loadings. Foam structure with a high porosity could not only lower the density but also greatly prolong the transmission path, enhancing the multiple reflection among cell walls and improving the EMI shielding ability [36–39]. Liang et al. [40] used wood-derived porous carbon (WPC) skeleton as a template to construct ultralight 3D MXene aerogel/WPC composites, which exhibited excellent EMI shielding properties at quite low density (71.3 dB, 0.197 g cm^−3^). However, the issues of high thickness, poor mechanical properties, and weak flexibility are still existing. Inspired by the structure of nacre, the “brick-and-mortar” structure with superimposed layer toughening effects could impart the composites with excellent toughness and strength [41–45]. For instance, Shahzad et al. [43] prepared nacre-like MXene/sodium alginate (SA) via vacuum-assisted filtration beginning from colloidal solutions. The 8-μm-thick flexible MXene/SA composite paper at 10 wt% SA content, in particular, exhibited extraordinary EMI SE of 57 dB. Moreover, the research results of Hu et al. [46] confirmed layer-by-layer structures were effective to integrate the individual advantages of components. The sandwich structured film based on Ca ion cross-linked SA-montmorillonite (MMT) and Ti_3_C_2_T_*x*_ MXene through step-by-step vacuum filtration simultaneously possessed effective EMI SE (50.01 dB), dramatically enhanced tensile strength (84.4 MPa), and fascinating fire resistivity.

However, along with the miniaturization and integration of modern electronics, some special components such as signal transmitters not only need to emit effective signal but also are protected from the external undesirable EM waves. Therefore, particular EMI shielding materials that are featured with different shielding performances, when EM waves are incident from different sides, are highly desirable for electronic devices. Latterly, gradient structures in asymmetric networks were extensively constructed to adjust the EMI shielding properties [47, 48]. Duan et al. [49] synthesized ordered multilayer gradient network composed of top hollow Ag-coated expanded polymer bead (EBAg) conductive shielding layer and bottom FeCo@rGO impedance matching layer. The EBAg/FeCo@rGO/WPU foams exhibited greatly slashed reflection coefficient (*R*) when EM waves were incident from FeCo@rGO layer. However, nearly equal total EMI SE values (SE_T_) were obtained for the foams at different incident sides. The gradient structures are of great significance for controlling its contribution from absorption and reflection, rather than the SE_T_. To date, no directional EMI shielding materials (i.e., the SE_T_ is quite different, when the EM wave is incident from different sides of the sample) have been reported in the literature.

In this paper, Ni-coated melamine foam (Ni@MF)/multiwalled carbon nanotube (CNT)/Poly (butylene adipate-co-terephthalate) (PBAT) composites with novel step-wise asymmetric structures were successfully fabricated via a facile solution encapsulation approach. Step-wise asymmetric Ni@MF/CNT/PBAT composites composed of loose porous Ni@MF layer (top) and tight CNT layer (bottom) definitely exhibit distinctly asymmetric electrical conductivity on the top and bottom surfaces. The slightly conductive Ni@MF layer on the top of composites serves as an EM absorption layer; the highly conductive CNT layer on the bottom plays a role as an EM reflection layer. As expected, step-wise asymmetric Ni@MF-5/CNT-75/PBAT composites reveal the novel directional EMI shielding performance with an obvious ΔSE_T_ of 8.8 dB. The directional EMI shielding of Ni@MF-5/CNT-75/PBAT composites is further confirmed by a practical application in a remote controlled toy car system. The step-wise asymmetric structure design strategy opens a new avenue for constructing and controlling directional EMI shielding materials for promising applications in next-generation communication technologies and smart intelligent electronics.

## Experimental Section

### Materials

Melamine foam (MF) sponges were purchased from Shanghai Beiyou Construction Materials Co., Ltd., China. Tin (II) chloride (SnCl_2_·2H_2_O), nickel chloride (NiCl_2_·6H_2_O), sodium citrate (C_6_H_5_Na_3_O_7_·2H_2_O), sodium hypophosphite (NaH_2_PO_2_), ammonium hydroxide (NH_3_·H_2_O), and polyethylene oxide (PEO, $\overline{{M_{{\text{v}}} }}$ ≈ 1.0 × 10^6^ g mol^−1^) were procured from Macklin Biochemical Co., Ltd., Shanghai, China. Palladium (II) chloride (PdCl_2_) was obtained from Sinopharm Chemical Reagent Co., Ltd., China. Hydrochloric acid (HCl, 37 wt%) was acquired from Chongqing Chuandong Chemical Co., Ltd., China. Multiwalled carbon nanotube (CNT, > 90%) with an external diameter of 10–20 nm and a length of < 30 μm was provided by Chengdu Organic Chemicals Co., Ltd., China. Dichloromethane (CH_2_Cl_2_, > 99.5%) was supplied by Tianjin Fuyu Fine Chemical Co., Ltd., China. Poly (butylene adipate-co-terephthalate) (PBAT) with a density of 1.21 g cm^−3^ and $\overline{{M_{{\text{w}}} }}$=1.42 × 10^5^ g mol^−1^ was achieved from Xinjiang Blue Ridge Tunhe Chemical Industry Joint Stock Co., Ltd., China.

### Preparation of Ni-Plated MF Sponges (Ni@MF)

Ni-plated MF sponges were prepared via a facile electroless plating process. Typically, commercially available MF sponges were cut into the dimension of 3.5 × 2.0 × 0.2 cm^3^ and cleaned with water in an ultrasonic washer for 3 times. Afterward, the MF sponges were sensitized in a 100 mL 2 wt% SnCl_2_/0.37 wt% HCl aqueous solution under ultrasonication for 30 min. The sensitized MF sponges were subsequently activated in a 100 mL activation solution comprising 10 mg PdCl_2_ and 0.1 mL HCl at ultrasonic treatment for 30 min. The activated MF sponges were rinsed with plenty of distilled water and dried. Then, 2 g NiCl_2_, 3 g C_6_H_5_Na_3_O_7_, and 12 mL NH_3_·H_2_O were dissolved into 90 mL deionized water to obtain the nickel-plating bath solution. The treated MF sponges were immersed in the plating bath, and a reducing agent (10 mL 40 wt% NaH_2_PO_2_ water solution) was added into the bath drop by drop with vigorous mechanical stirring at 50 °C. After a certain amount of plating time, the Ni@MF sponges were washed with distilled water and dried in a vacuum oven at 40 °C.

### Fabrication of CNT Papers

A facile vacuum-assisted self-assembly approach was proposed to fabricate CNT papers. In a typical procedure, 20 mg of PEO was dissolved into 200 mL deionized water with continuous stirring, and then, 200 mg CNT was dispersed into the obtained PEO solution under ultrasonication for 20 min. Thereafter, a certain quantity of CNT suspension was filtered in a vacuum by a cellulose acetate filter membrane with a diameter of 47 mm and pore size of 0.22 μm. Finally, CNT papers were peeled off from the filter membranes after drying at room temperature overnight. A series of CNT papers with different thicknesses of 25, 50, 75, and 100 μm were successfully fabricated via controlling the quantity of CNT suspension.

### Fabrication of Ni@MF/CNT/PBAT Composites

The Ni@MF/CNT/PBAT composites were manufactured by simple solution encapsulation. Firstly, 4 g of PBAT was dissolved into 10 mL CH_2_Cl_2_ under vigorous agitation, followed by degassing and casting into a culture dish. Afterward, Ni@MF and CNT paper were together immersed into the PBAT CH_2_Cl_2_ solution, where CNT paper was situated beneath Ni@MF. The culture dish was transferred in an oven at 60 °C for 12 h to evaporate the solvent, and Ni@MF/CNT/PBAT composites were finally obtained. For brevity, the prepared Ni@MF/CNT/PBAT composites were labeled as Ni@MF-*x*/CNT-*y*/PBAT, where *x* represents the nickel-plating time for Ni@MF (min) and *y* denotes the thickness of CNT paper (μm). In addition, the thickness of all the samples is 1.8 mm after tailoring.

### Characterizations

The morphology and microstructures of Ni@MF, CNT papers, and Ni@MF/CNT/PBAT composites were observed on field emission scanning electron microscope (FESEM, Hitachi SU8010, Japan). Energy-dispersive spectrum (EDS) mappings were applied to determine the distribution of Ni element in Ni@MF and composites. So as to investigate the crystalline structures, X-ray diffraction (XRD) patterns were recorded on a PANalytical diffractometer (the Netherlands) with Cu K*α* radiation (*λ* = 1.5406 Å) in the range from 5° to 90°. The surface element states of samples were estimated by using an X-ray photoelectron spectrometer (XPS, Escalab 250XI, Thermo Fisher Scientific, USA). The volume resistivity of composites was tested using a four-point probe resistivity determiner (Signatone, USA). The probe was penetrated into the CNT layer or Ni@MF layer of Ni@MF/CNT/PBAT composites during the measurement. A vector network analyzer (VNA, N5244A, Agilent Technologies, USA) was employed to detect the electromagnetic interference (EMI) shielding effectiveness (SE) of such composites over the X-band (8.2–12.4 GHz). From the scattering parameters, S_11_ and S_21_, the power coefficient of transmissivity (T), absorptivity (A), and reflectivity (R), the total EMI SE (SE_T_), reflection EMI SE (SE_R_), and absorption EMI SE (SE_A_) were calculated by Eqs. (1–8) [15, 50]:

1 $$R = \left| {S_{11} } \right|^{2} \,{\text{and}}\,T = \left| {S_{21} } \right|^{2}$$

2 $$A = 1 - \left( {T + R} \right)$$

3 $${\text{SE}}_{{\text{T}}} = 10\log \frac{1}{T}$$

4 $${\text{SE}}_{{\text{R}}} = 10\log \left( {\frac{1}{1 - R}} \right)$$

5 $${\text{SE}}_{{\text{A}}} = 10\log \left( {\frac{1 - R}{T}} \right)$$

6 $${\text{SE}}_{{\text{T}}} = {\text{SE}}_{{\text{R}}} + {\text{SE}}_{{\text{A}}} + {\text{SE}}_{{\text{M}}}$$

7 $${\text{SE}}_{{\text{T}}} = \left| {{\text{SE}}_{{{\text{T1}}}} - {\text{SE}}_{{{\text{T2}}}} } \right|$$

8 $${\text{SE}} \,{\text{enhancement}} = {\raise0.7ex\hbox{${\Delta {\text{SE}}_{{\text{T}}} }$} \!\mathord{\left/ {\vphantom {{\Delta {\text{SE}}_{{\text{T}}} } {{\text{SE}}_{{{\text{T2}}}} }}}\right.\kern-\nulldelimiterspace} \!\lower0.7ex\hbox{${{\text{SE}}_{{{\text{T2}}}} }$}}$$

where “1” and “2” denote that the EM wave is incident from Ni@MF layer and CNT layer, respectively.

When SE_T_ is greater than 15 dB, SE_M_ could be usually ignored. Tensile measurement was conducted on a universal testing machine (Model 5576, Instron, USA) with a loading rate of 1 mm min^−1^ at ambient temperature.

## Results and Discussion

### Fabrication of Step-Wise Asymmetric Conductive Networks

**Fig. 1:**
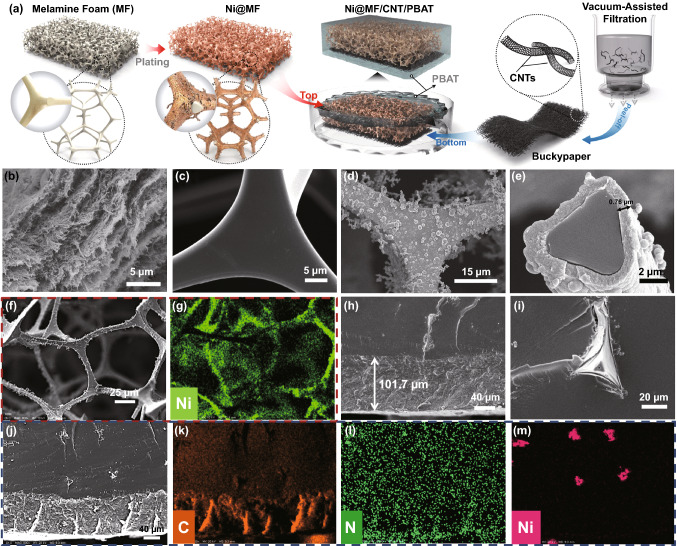
Fabrication of step-wise asymmetric Ni@MF/CNT/PBAT composites. **a** Schematic diagram for the preparation process of step-wise asymmetric Ni@MF/CNT/PBAT composites. **b** and **c** SEM images of CNT paper and pure MF sponge, respectively. **d** and **e** SEM images of Ni@MF-5 at different magnifications, where 5 represents the Ni-plating time of 5 min. **f** and **g** SEM of Ni@MF-5 with Ni elemental EDS mapping. **h** SEM image of the lower CNT-75/PBAT layer in Ni@MF-5/CNT-75/PBAT, where 75 denotes the thickness of CNT paper (μm). **i** SEM image of the upper Ni@MF-5/PBAT layer. **j-m** SEM of Ni@MF-5/CNT-75/PBAT with C, N, Ni elemental EDS mappings

The meaningful design of step-wise asymmetric Ni@MF/CNT/PBAT composites involves the vacuum-assisted self-assembly of CNT paper, electroless Ni plating for MF, and their integrated encapsulation in PBAT dichloromethane solution (Figs. 1a and S1). During the vacuum-assisted filtration process of CNT paper, a bit of polyethylene oxide (PEO), which is water-soluble, environmentally benign, as well as compatible with CNT, is specially introduced to cohere neighbor CNTs, guaranteeing excellent compactibility of CNT paper. Thus, CNT paper definitely exhibits the pretty aligned lamellar microstructures along in-plane direction with slight undulation, on account of the intense interaction between CNTs and PEO such as hydrogen bonds (Fig. 1b). CNTs are compactly stacked and entangled together to build the tight connected conductive network during the vacuum filtration, contributing to the superior electrical conductivity of CNT paper [50, 51]. SEM images of pure MF reveal that MF sponge possesses a perfect 3D porous structure (Fig. S2a) and the surface of MF skeleton is quite smooth without any protrusion (Fig. 1c). After 5 min of Ni plating, Ni@MF-5 exhibits a rough skeleton surface (Fig. 1d) and tiny Ni particles are tightly clustered together to form a uniform Ni layer of 0.76 μm thickness (Figs. 1e and S3), whereas the 3D porous structure is well remained (Fig. S2b). Furthermore, SEM of Ni@MF-5 with Ni elemental EDS mapping (Fig. 1f, g) illuminates that Ni element is mainly selectively distributed on the MF cell walls, further confirming the successful electroless plating of Ni particles. The morphology of Ni@MF is greatly dependent on the plating time. Generally, more Ni particles are deposited on the MF skeleton and the increasingly tough surface is consequently obtained with the prolonged plating time (Fig. S4). The loading of Ni layer is gradually elevated from 22.1 wt% for 1 min plating time to 73.9 wt% for 5 min plating time (Fig. S5). These results certainly illustrate that compact CNT paper and loose Ni@MF with superb conductive networks are successfully yielded.

Afterward, CNT paper and Ni@MF are impregnated together into PBAT dichloromethane solution to fabricate step-wise asymmetric Ni@MF/CNT/PBAT composites. Ni@MF-5/CNT-75/PBAT (Ni@MF-*x*/CNT-*y*/PBAT: *x* is the plating time (min), and *y* represents the thickness of CNT paper (μm)) displays the evident step-wise layered structures (Fig. 1h). The fracture surface of the upper Ni@MF-5/PBAT layer is a lot smoother, except some MF skeleton protrusions are exposed (Fig. 1i). However, a much rougher fracture surface is clearly observed for the lower CNT-75/PBAT layer with a thickness around 101.7 μm which is higher than that of CNT paper before impregnation (Fig. 1h). The EDS mappings of the sample can help in validating the construction of step-wise asymmetric networks (Fig. 1j–m). The C element is more densely distributed in lower CNT/PBAT layer, while the distribution of Ni element is merely accumulated at the exposed Ni@MF skeletons in upper Ni@MF/PBAT layer. For various Ni@MF foams with different plating times, Ni@MF/CNT/PBAT composites can be all facilely prepared, and the plating time has little impact on the step-wise asymmetric structure (Fig. S6). Besides, Ni@MF/CNT/PBAT composites with different thicknesses of CNT paper also exhibit the unique step-wise asymmetric structure (Fig. S7). The thickness of the lower CNT/PBAT layer is continuously increased with the incremental thickness of CNT paper, ranging from 25.2 μm for Ni@MF-3/CNT-25/PBAT to 127.1 μm for Ni@MF-3/CNT-100/PBAT (Fig. S8). Hence, the step-wise asymmetric structure is able to be adjusted to some extent by changing the fabrication condition.

### Structures of Ni@MF/CNT/PBAT Composites

**Fig. 2:**
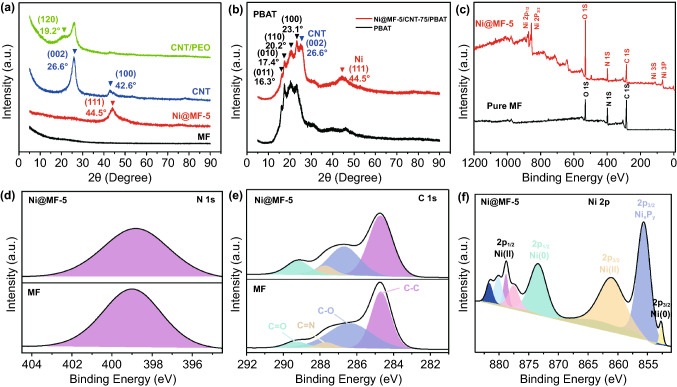
Structure characterization of samples. **a** XRD patterns of MF, Ni@MF-5, CNT, and CNT/PEO. **b** XRD patterns of PBAT and Ni@MF-5/CNT-75/PBAT. **c** Full-range XPS spectra of MF and Ni@MF-5. **d** Narrow N 1 s core-level XPS spectra of MF and Ni@MF-5. **e** Narrow C 1 s core-level XPS spectra of MF and Ni@MF-5. **f** Narrow Ni 2p core-level XPS spectrum of Ni@MF-5

XRD analysis is employed to determine the phase of composite structures. It is conspicuously found that MF as an uncrystallized polymer exhibits a typical amorphous XRD pattern (Fig. 2a). After the plating of Ni, the visible characteristic diffraction peak for Ni@MF-5 at 44.5° is assigned to the (111) plane of face-centered cubic phase Ni, illustrating that well-crystallized FCC Ni nanoparticles are formed on the pore wall of MF sponges (Fig. 2a). Two conspicuous diffraction peaks observed at 26.6° and 42.6° in CNT XRD curve are indexed to (002) and (100) lattice planes of CNT crystalline structures, respectively. Besides, a new moderate peak is located at 19.2° for CNF/PEO paper, corresponding to the (120) plane of PEO. PBAT, a semi-crystalline polymer, exhibits the four strong peaks at 16.3°, 17.4°, 20.2°, and 23.1°, which is in accord with (011), (010), (110), and (100) lattice planes of PBAT crystals (Figs. 2b and S9). Strikingly, all the above featured peaks are also clearly traced in the XRD pattern of Ni@MF-5/CNT-75/PBAT, which indicates that the encapsulation has little effect on the crystal structures of the constituents (Fig. 2b).

The chemical composition and surface element state of Ni@MF-5 are detected by XPS characterization. The full XPS pattern of pure MF manifests the existence of C, N, and O elements, whereas the survey spectrum of Ni@MF-5 reveals the successful deposition of zero-valent Ni (Fig. 2c and Table S1). The characteristic peaks corresponding to Ni2p_1/2_, Ni2p_3/2_, Ni3s, and Ni 3p are detailedly located at the binding energy of 872.1, 855.3, 112.5, and 67.5 eV, respectively. The N 1 s core-level peaks of both MF and Ni@MF-5 are centered at 398.8 eV; however, the peak width is intuitionally raised with the conversion plating of Ni (Fig. 2d), which signifies the excellent interaction between MF and Ni particles. In the high-resolution C 1 s XPS spectra (Fig. 2e), four major feature peaks situated at 289.2, 287.6, 286.5, and 284.8 eV for both MF and Ni@MF-5 are attributed to –C=O, C=N, C–O, and C–C, respectively. Therefore, the chemical structures of MF are well reserved after the electroless plating of Ni. The Ni 2p core-level spectrum of Ni@MF-5 can be appropriately fitted with two types (Ni 2p_1/2_ and Ni 2p_3/2_, Fig. 2f), which is consistent with the results of Fig. 2c. In detail, Ni 2p_1/2_ at 873.5 eV and Ni 2p_3/2_ at 852.7 eV are ascribed to metallic Ni (Ni^0^), indicating that some Ni^2+^ ions have been reduced to Ni^0^ valence state during the Ni plating. Furthermore, Ni 2p_1/2_ at 881.6–878.8 eV and Ni 2p_3/2_ at 861.2 eV are corresponding to Ni^2+^ in the form of NiO and Ni(OH)_2_, due to the introduction of oxygen groups during the deposition process and/or the surface oxidation of Ni nanoparticles by air. The sharp Ni 2p_3/2_ component at 855.7 eV is assigned to Ni_x_P_y_, which means that Ni and P participate in the form of Ni–P compounds in the plating procedure [52–54]. On the basis of these results, it can be concluded that Ni nanoparticles are successfully coated on the MF sponges.

### Directional EMI Shielding

Generally, the electrical conductivity of materials is of great relevance to the EMI shielding performance [16, 42, 55]. It can be proved that the electrical conductivity of Ni@MF foams is enormously elevated from 28.6 to 460.8 S m^−1^, with the plating time extending from 1 to 5 min (Fig. S10a). All the CNT papers exhibit excellent electrical conductivity on the magnitude of 10^3^ S m^−1^ (Fig. S10b), owing to the continuous conductive networks built by the entangled and connected CNTs. Although the bottom electrical conductivity of Ni@MF/CNT/PBAT composites is evidently decreased after the encapsulation because of the poor conductivity of PBAT matrix, the bottom electrical conductivity is yet satisfactory with the highest value of 469.6 S m^−1^ for Ni@MF-3/CNT-100/PBAT, which far surpasses the basic requirement (1 S m^−1^) for practical applications of conductive EMI shielding materials (Fig. S11) [56]. However, the top surfaces of Ni@MF/CNT/PBAT composites are electrically insulating (< 10^–8^ S m^−1^), due to the physical isolation of PBAT. Thus, the top and bottom surfaces of Ni@MF/CNT/PBAT composites display the distinct electrical conductivity, which is in accordance with the structures from SEM images.

**Fig. 3:**
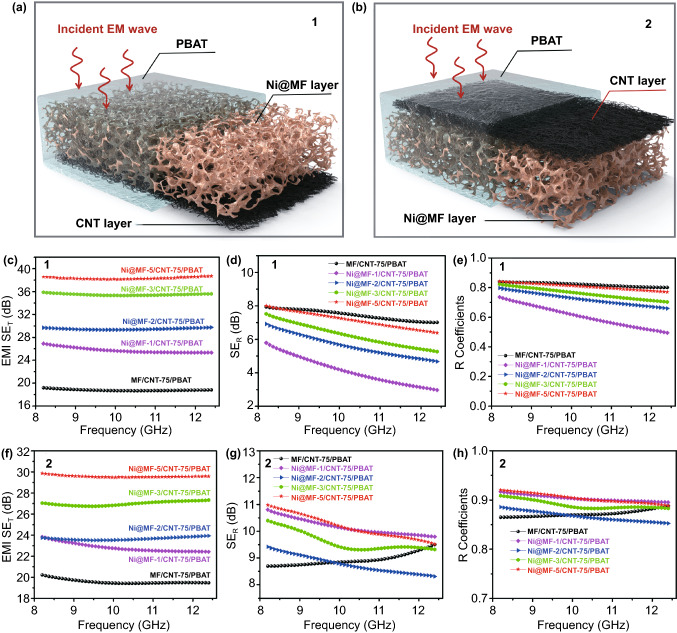
Directional EMI shielding performances. **a** Schematic for experiment 1, the incident EM wave from Ni@MF layer. **b** Schematic for experiment 2, the incident EM wave from CNT layer. **c** EMI SE_T_, **d** SE_R_, and **e** R coefficients in X-band for Ni@MF/CNT-75/PBAT composites, when the EM wave is incident from Ni@MF layer (experiment 1). **f** EMI SE_T_, **g** SE_R_, and **h** R coefficients in X-band for Ni@MF/CNT-75/PBAT composites, when the EM wave is incident from CNT layer (experiment 2)

**Fig. 4:**
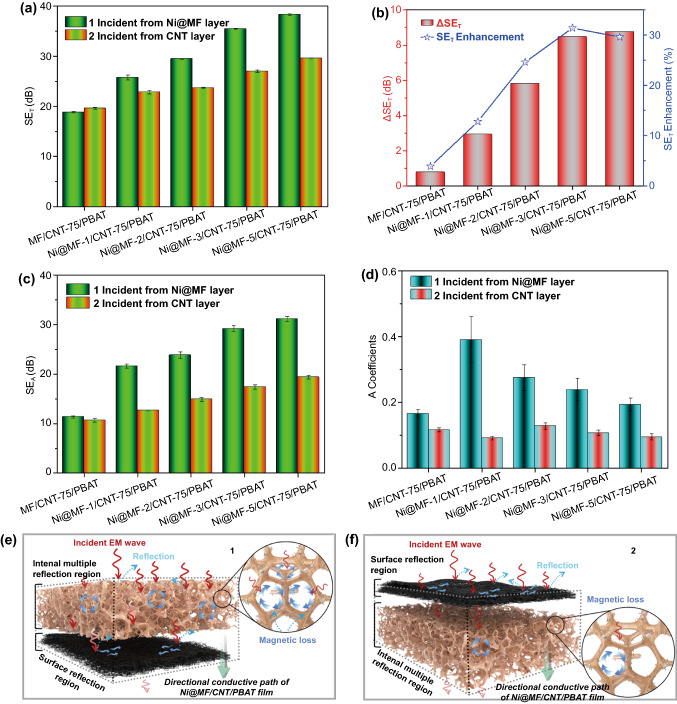
Directional EMI shielding mechanism. **a** The average SE_T_ of Ni@MF/CNT-75/PBAT composites with the incident EM wave from different sample sides. **b** ΔSE_T_ and SE_T_ enhancement of Ni@MF/CNT-75/PBAT composites when the EM wave is incident from different sample sides. **c** The average SE_A_ and **d** coefficients of Ni@MF/CNT-75/PBAT composites with the incident EM wave from different sample sides. **e, f** Schematic diagram of the directional EMI shielding mechanism for step-wise asymmetric Ni@MF/CNT-75/PBAT composites

As expected from the superb electrical conductivity and step-wise asymmetry, Ni@MF/CNT/PBAT composites exhibit the unprecedented directional EMI shielding performance. Experiments “1” and “2” indicate that the EM wave is incident from Ni@MF layer and CNT layer, respectively (Fig. 3a, b). Regardless of the different incident directions, the EMI SE_T_ of all the composite samples reveals weak frequency dependence over the whole X-band (Fig. 3c, f). In addition, the EMI SE_T_ values are significantly enhanced with the increasing plating time, following a parallel tendency to the varying electrical conductivity. MF/CNT-75/PBAT displays the lowest EMI SE_T_ around 20 dB and the similar values (SE_T1_ and SE_T2_) at different incident directions of the EM wave (Figs. 3c, f and 4a). The EMI shielding performance of MF/CNT-75/PBAT mainly stems from the conductive CNT layer, due to the insulating pure MF. The EMI SE_T1_ of Ni@MF-5/CNT-75/PBAT is enhanced up to the optimal value of 38.3 dB, when the EM wave is incident from Ni@MF-5 layer (Fig. 4a). Strikingly, the EMT SE_T2_ of Ni@MF-5/CNT-75/PBAT with the incident EM wave from CNT layer is only 29.5 dB, which is much lower than the EMI SE_T1_. Furthermore, Ni@MF-5/CNT-75/PBAT exhibits ΔSE_T_ of 8.8 dB and SE enhancement of 30%, indicating the novel directional EMI shielding property (Fig. 4b).

To theoretically clarify the directional EMI shielding mechanism of step-wise asymmetric Ni@MF/CNT-75/PBAT composites, the absorption SE (SE_A_), reflection SE (SE_R_), reflectivity power coefficient (R), and absorptivity power coefficient (A) were calculated according to Eqs. (1–6). It is obviously seen that the largest SE_R1_ and R_1_ are both obtained for MF/CNT-75/PBAT, when the EM wave is incident from Ni@MF layer (Fig. 3d, e). Since pure MF layer is almost transparent to EM wave, a majority of incident EM wave passes through MF layer with little attenuation. Then, a large part of EM wave is reflected back at the MF-CNT layer interface owing to their impedance mismatch, which is closely related to the existence of plentiful mobile charge carriers in conductive CNT networks. For Ni@MF/CNT-75/PBAT composites, both SE_R1_ and R_1_ illustrate the visually ascending tendency with the incremental plating time, which is in accord with the impedance mismatch theory [57]. Particularly, the SE_R1_ and R_1_ values of Ni@MF-5/CNT-75/PBAT composite are 7.2 dB and 0.81, respectively (Fig. S12). At the incident EM wave from CNT-75 layer, the SE_R2_ and R_2_ of Ni@MF/CNT-75/PBAT composites display the inapparent and irregular variation with the extending plating time (Figs. 3g, h, and S12). When the EM wave comes across the CNT-75 layer, enormous reflection immediately occurs due to the impedance mismatch between air and the conductive CNT layer, which mainly determines the SE_R2_ and R_2_ values. Thus, the SE_R2_ and R_2_ values of Ni@MF/CNT-75/PBAT composites have little dependence on the plating time. Comparatively, SE_R2_ and R_2_ are clearly higher than SE_R1_ and R_1_, respectively, for the identical Ni@MF/CNT-75/PBAT composite, which is ascribed to the far higher electrical conductivity of bottom CNT layer than that of top Ni@MF layer. Detailedly, the ΔSE_R_ and SE_R_ decrease of Ni@MF-1/CNT-75/PBAT is as high as 6.1 dB and 59%, respectively, which are the maximum values of Ni@MF/CNT-75/PBAT composites (Fig. S13).

At different incident directions, both SE_A1_ and SE_A2_ of Ni@MF/CNT-75/PBAT composites are significantly prompted after prolonging the plating time, which is attributed to the increasing magnetic Ni particles and dissipative moving charge carriers (Fig. S14). A_1_ reveals an inverse change tendency to R_1_ with the incremental plating time (Fig. S15a). Apparently, Ni@MF-1/CNT-75/PBAT displays the maximum A_1_ of 0.39 but the minimum R_1_ of 0.61 among the Ni@MF/CNT-75/PBAT composites (Fig. 4d). Like R_2_, A_2_ exhibits stable fluctuation with the varying plating time, on account of the superb electrical conductivity of CNT-75 layer (Figs. 4d and S15b). In addition, SE_A1_ and A_1_ are absolutely superior to SE_A2_ and A_2_, respectively, for a single Ni@MF/CNT-75/PBAT sample (Fig. 4c, d), which illuminates that the step-wise asymmetric structure plays a crucial role in manipulating not only the total EMI SE but also the contributions from reflection and absorption. For example, Ni@MF-5/CNT-75/PBAT possesses the high SE_A1_ of 31.2 dB and the low SE_A2_ of 19.4 dB (ΔSE_A_ = 11.8 dB and SE_A_ enhancement = 61%, Fig. S16). Thus, in comparison with SE_T2_, the SE_T1_ enhancement is primarily ascribed to the enormous improvement of microwave absorption.

To better make sense of the directional shielding mechanism, the EM wave transfer across step-wise asymmetric Ni@MF/CNT-75/PBAT composites at different incident directions is schematically illustrated in Fig. 4e, f. No matter which side of Ni@MF/CNT-75/PBAT composites EM wave is incident from, R is always higher than A, indicating the dominant role of EM reflection in the EMI shielding process. This phenomenon is mainly ascribed to the fact that a large part of EM wave is reflected by the material surface before penetrating it [42]. When the EM wave is incident from Ni@MF layer, some incident EM wave passes into Ni@MF layer with relatively weak reflectivity due to the low electrical conductivity of Ni@MF layer (Fig. 4e). The entered EM wave is trapped in the porous Ni@MF sponge and intensively interacts with the high-density charge carriers. Consequently, a large part of EM wave is absorbed via magnetic and dielectric losses [58]. Furthermore, the multiple reflection of EM wave in pores can prolong the propagation path to promote the absorption or dissipation of EM wave [59]. As the penetrated EM wave encounters the high conductive layer of CNT-75, most of the remaining EM wave is reflected back into the porous Ni@MF layer, due to the large impedance mismatch between Ni@MF and CNT-75 layers. The reflected EM wave is highly absorbed again via the above-mentioned magnetic loss, dielectric loss, and multiple reflection in porous Ni@MF sponge, thus effectively raising the SE_A1_ value. A spot of reflected EM wave will ultimately propagate back into space after the dissipation in Ni@MF sponge, which is able to offset the weak reflection (SE_R1_) to some extent. Afterward, the EM wave entered CNT-75 layer is further attenuated, owing to interacting with free charge carriers in the conductive networks. The reasonable step-wise asymmetric structures give rise to “weak reflection–absorption–strong reflection–reabsorption” mechanism when EM wave is incident from the Ni@MF layer. In this manner, the composites exhibit wonderful EMI SE against the EM wave. At the incident direction from CNT-75 layer, the high conductive CNT layer directly reflects a large portion of the incident EM wave before allowing it to enter, by reason of the great impedance mismatch (Fig. 4f). Hence, the subsequent absorption in porous Ni@MF sponge is largely reduced. Additionally, the second reflection at the interface between Ni@MF layer and air is too weak to evidently contribute to the total EMI shielding, because of the low electrical conductivity of Ni@MF layer. It can be proved that the EM incident from CNT-75 layer encounters the “strong reflection–absorption” process, and thus, the energy loss is obviously lower than that incident from Ni@MF layer. We can draw a conclusion that step-wise asymmetric Ni@MF/CNT-75/PBAT composites exhibit unprecedented directional EMI shielding performances and may open up a new research direction for EMI shielding materials.

So as to evaluate the effect of CNT thickness, the EMI shielding performances of Ni@MF-3/CNT/PBAT composites with various CNT thickness were systemically characterized (Figs. S17–S19). The incident direction of EM wave is from the Ni@MF-3 layer. It is easily found that SE_T_ of Ni@MF-3/CNT/PBAT composites is enhanced with the incremental thickness of CNT paper, which is independent from frequency. Note that the SE_T_ value is clearly raised from 19.9 dB for Ni@MF-3/PBAT to 35.9 dB for Ni@MF-3/CNT-100/PBAT. The SE_A_ of Ni@MF-3/CNT/PBAT composites also exhibits the rising trend with the increase of CNT thickness, whereas SE_R_ of the composites shows no obvious variation. This illustrates that the enhancement of SE_T_ is mainly stemming from the improved microwave absorption. The SE_A_ and SE_R_ of Ni@MF-3/CNT-100/PBAT are 30.4 and 5.5 dB, respectively. Furthermore, the A coefficient continuously increases, but the R coefficient slightly decreases, as the thickness of CNT paper is elevated. Ni@MF-3/CNT-100/PBAT exhibits the largest A of 0.28 and lowest R of 0.72. This exciting phenomenon can be ascribed to the rational step-wise asymmetric structures. The thicker CNT layer with higher conductivity (Fig. S11) can reflect more incident EM back into porous Ni@MF layer where the reflected EM can be further reabsorbed or dissipated in the form of heat via magnetic loss and dielectric loss [60]. As a result, the step-wise asymmetric Ni@MF/CNT/PBAT composites have great potential for efficient EMI shielding in portable and intelligent electronics.

### Practical Application for Directional EMI Shielding

**Fig. 5:**
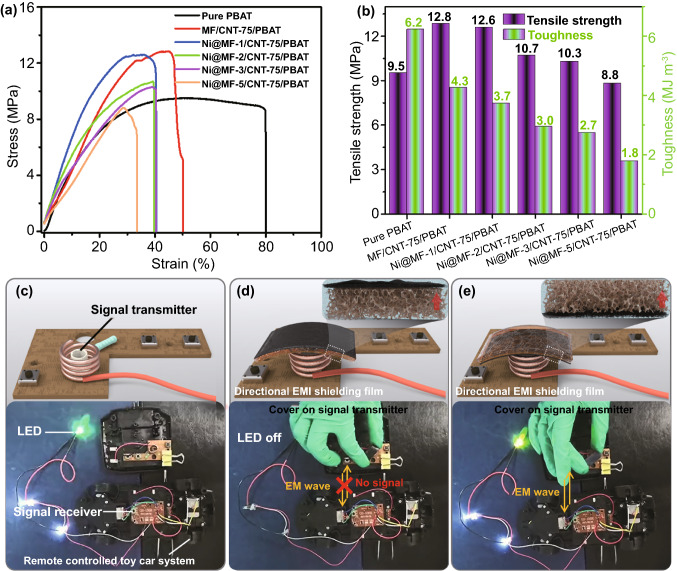
Practical application for directional EMI shielding. **a** Typical stress–strain curves of pure PBAT and step-wise asymmetric Ni@MF/CNT-75/PBAT composites with different Ni-plating times. **b** The corresponding tensile strength and toughness. **C–e** A real directional EMI shielding application measurement in a remote controlled toy car system. The signal transmitter is faced with **c** no shielding material, **d** Ni@MF layer, and **e** CNT-75 layer of the Ni@MF-5/CNT-75/PBAT composite

As highly effective EMI shielding materials, fantastic mechanical performances are also of great significance for the practical application, especially in the fields of portable and wearable electronic devices. Herein, tensile measurements were conducted to explore the mechanical properties of Ni@MF/CNT-75/PBAT composites. The typical stress–strain curves of Ni@MF/CNT-75/PBAT composites reveal that all the composites are fractured in a ductile manner with satisfactory strain, by reason of the flexible PBAT chains, which is vital for enduring mechanical deformation (Fig. 5a). Specially, pure PBAT exhibits a tensile strength of 9.5 MPa, a toughness of 6.2 MJ m^−3^, a Young’s modulus of 33.2 MPa, and a fracture strain of 80.1% (Figs. 5b and S20, S21). With the introduction of rigid MF, the tensile strength and Young’s modulus of the MF/CNT-75/PBAT composite are significantly enhanced to 12.8 and 44.2 MPa, respectively, due to the excellent strengthening effect of MF. Correspondingly, the reduced toughness of 4.3 MJ m^−3^ and strain at break of 50.1% are achieved for MF/CNT-75/PBAT composite. Besides, all the crucial tensile properties regarding tensile strength, toughness, Young’s modulus, and fracture strain of Ni@MF/CNT-75/PBAT composites are deteriorated with the increasing plating time. The most probable reason is that the rough Ni nanoparticles decorated on MF sponge induce more defect (void) and stress concentration points that require less energy to propagate the cracks. Fortunately, Ni@MF/CNT-75/PBAT composites yet present the acceptable mechanical properties for the actual application in portable microelectronics.

In order to intuitionally illustrate the novel directional EMI shielding performances of step-wise asymmetric Ni@MF/CNT/PBAT composites, a real application test was conducted using a remote controlled toy car system consisting of a motor module, indicator module, transmitter module, and receiver module (Fig. 5c–e and Video S1). The signal transmitter with a functional voltage of 3 V sends the signal microwave that can be received by the receiving module. Then, the indicator lights are flashed, and the motor is brought into operation (Fig. 5c and Video S1). When the signal transmitter is faced with the Ni@MF layer of Ni@MF-5/CNT-75/PBAT, that is, the EM is incident from Ni@MF layer into the composite, the indicator lights are switched off and the motor stops running (Fig. 5d). This is attributed to the fact that the signal microwave is efficiently blocked by Ni@MF-5/CNT-75/PBAT with the incident direction from Ni@MF layer. However, the indicator lights are able to be turn on again and the motor also is set into motion by turning over the step-wise asymmetric Ni@MF-5/CNT-75/PBAT, where the signal microwave is incident from the CNT-75 layer. This interesting phenomenon can provide a new strategy for designing the step-wise asymmetric shielding composites in the creative field of directional EMI shielding.

## Conclusions

In this work, we successfully designed step-wise asymmetric conductive networks to fabricate the novel directional EMI shielding composite materials. Ni@MF sponges were obtained by a facile electroless plating process, and CNT papers were prepared via a simple vacuum-assisted self-assembly approach. Then, solution encapsulation was applied to fabricate Ni@MF/CNT/PBAT composites with step-wise asymmetric structures. Ni@MF/CNT/PBAT composites display the distinctly asymmetric electrical conductivity on the top and bottom surfaces, in good agreement with the structure analysis from SEM images. As expected from the superb electrical conductivity and step-wise asymmetric structures, Ni@MF/CNT/PBAT composites exhibit the extraordinary directional EMI shielding performance. When the EM wave is incident from Ni@MF layer, the EMI SE_T1_ of Ni@MF-5/CNT-75/PBAT is elevated up to the optimal value of 38.3 dB with the prolonged plating time. Strikingly, at the incident EM wave from CNT paper, the EMT SE_T2_ of Ni@MF-5/CNT-75/PBAT is only 29.5 dB, showcasing the obvious ΔSE_T_ of 8.8 dB and SE enhancement of 30%. The reasonable step-wise asymmetric structures consisting of loose Ni@MF and compact CNT layers can produce a special “weak reflection–absorption–strong reflection–reabsorption” course for the incident EM wave from Ni@MF layer. In a practical directional EMI shielding application, Ni@MF-5/CNT-75/PBAT composite is proved to efficiently block the signal microwave transmission, when the signal microwave is incident from Ni@MF layer. If turning over Ni@MF-5/CNT-75/PBAT composite, the remote controlled toy car system is brought into operation once again, which demonstrates the directional EMI shielding of Ni@MF/CNT/PBAT composites in actual applications. This investigation provides a significant insight into the construct and fabrication of step-wise asymmetric polymeric composites with novel directional EMI shielding properties for the enormous prospect in portable electronics and communication industry.

## Supporting Information

SEM images of pure MF, Ni@MF, and Ni@MF/CNT/PBAT composites; Ni percentage in Ni@MF; The thickness of CNT/PBAT layer in Ni@MF-3/CNT/PBAT composites; The magnified XRD pattern of PBAT in 10–30°; Resistance and conductivity of Ni@MF, CNT paper, and Ni@MF/CNT/PBAT composites; EMI shielding properties of Ni@MF/CNT/PBAT composites; Young’s modulus and fracture strain of Ni@MF/CNT-75/PBAT composites.

## Supplementary Information

Below is the link to the electronic supplementary material.

Supplementary file1 (PDF 1935 kb)

Supplementary file2 (MP4 1761 kb)
