# Supplementary material for: Directional Electromagnetic Interference Shielding Based on Step-Wise Asymmetric Conductive Networks
Source: Nanomicro Lett. 2021 Dec 6;14:16. doi: 10.1007/s40820-021-00743-y (PMC8648885; doi:10.1007/s40820-021-00743-y)
Supplement: Supplementary file 1 — Supplementary file1 (PDF 1935 kb) [file 40820_2021_743_MOESM1_ESM.pdf]

Supporting Information for

Directional Electromagnetic Interference Shielding Based on Step-Wise Asymmetric Conductive Networks

Bai Xue[1, 2], Yi Li[1], Ziling Cheng[1], Shengdu Yang[1], Lan Xie[1, 2, *], Shuhao Qin[2], Qiang Zheng[1, 3, 4, *]

[1]Department of Polymer Materials and Engineering, College of Materials and Metallurgy, Guizhou University, Guiyang 550025, P. R. China

[2]National Engineering Research Center for Compounding and Modification of Polymer Materials; National and Local Joint Engineering Research Center for Functional Polymer Membrane Materials and Membrane Processes, Guiyang 550014, P. R. China

[3]College of Polymer Science and Engineering, Zhejiang University, Hangzhou 310027, P. R. China

[4]College of Materials Science and Engineering, Taiyuan University of Technology, Taiyuan, 030024, P. R. China

*Correspondence authors. E-mail: mm.lanxie@gzu.edu.cn (L. X.) or zhengqiang@zju.edu.cn (Q. Z.)

Supplementary Figures and Table

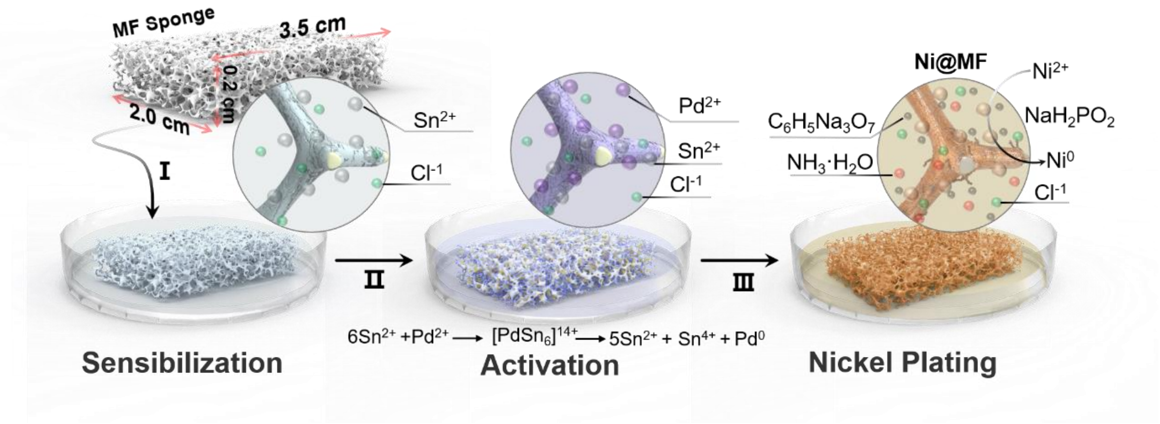

Fig. S1 Schematic diagram for the syntheses of Ni@MF

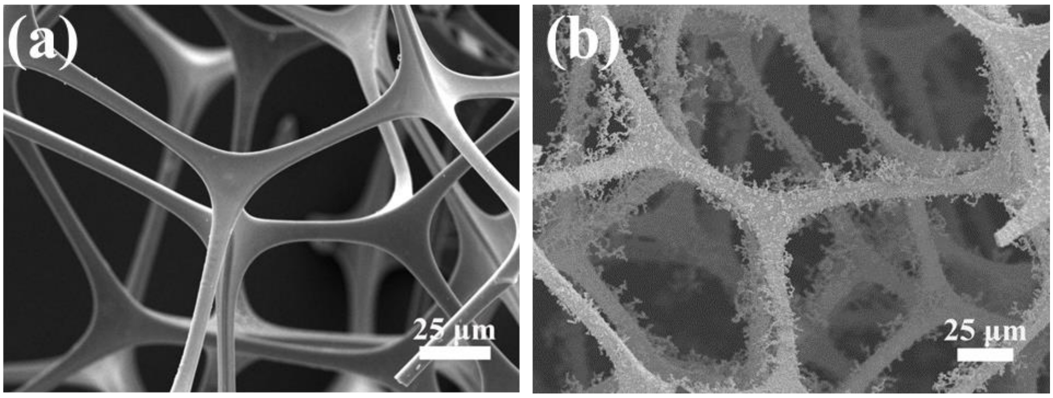

Fig. S2 SEM images of (**a**) pure MF and (**b**) Ni@MF-5

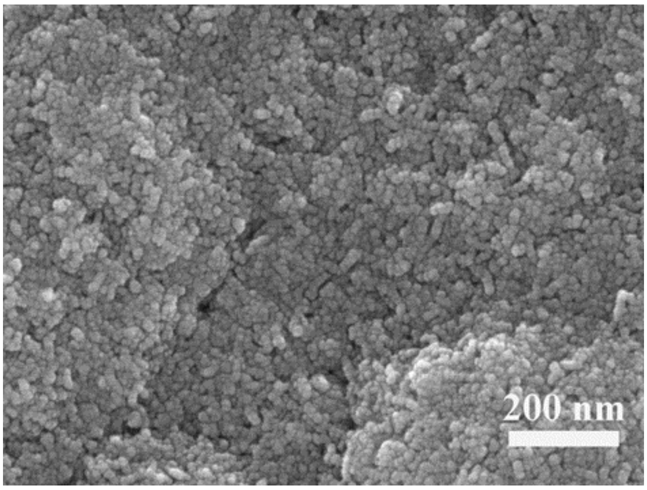

Fig. S3 SEM image of Ni@MF-5 at high magnification

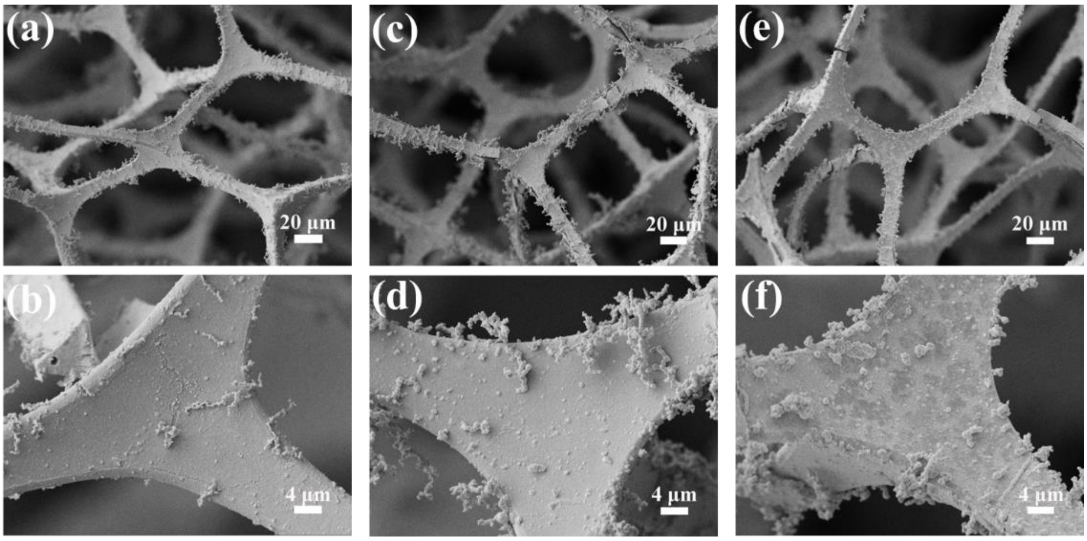

Fig. S4 SEM images of Ni@MF with different plating time. SEM images of (**a** and **b**) Ni@MF-1, (**c** and **d**) Ni@MF-2, and (**e** and **f**) Ni@MF-3

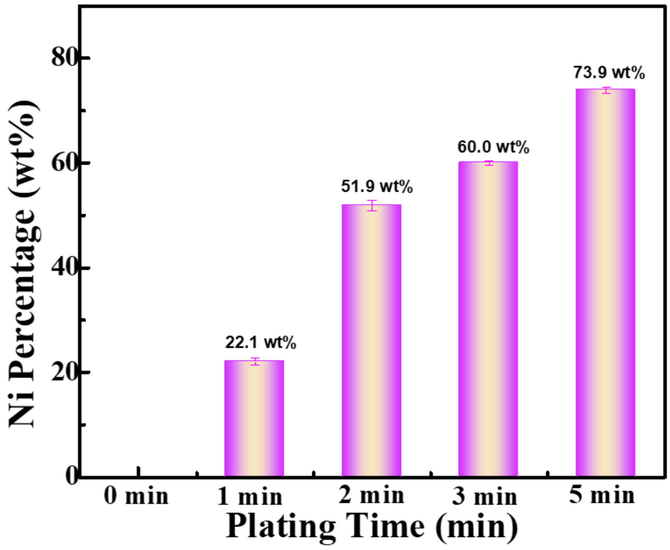

Fig. S5 Ni percentage in Ni@MF as a function of plating time

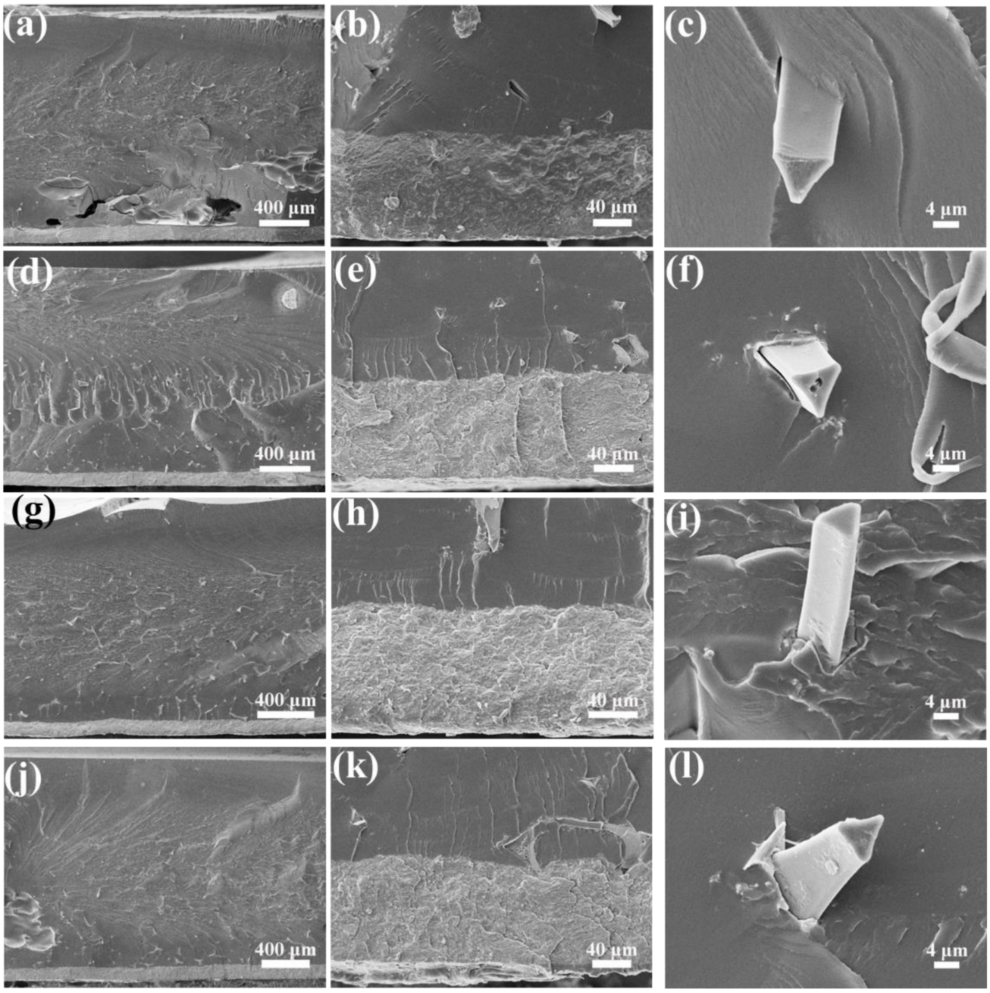

Fig. S6 SEM images of Ni@MF/CNT-75/PBAT composites with different Ni-plating time. SEM images of (**a-c**) MF/CNT-75/PBAT composites, (**d-f**) Ni@MF-1/CNT-75/PBAT composites, (**g-i**) Ni@MF-2/CNT-75/PBAT composites, and (**j-l**) Ni@MF-3/CNT-75/PBAT composites

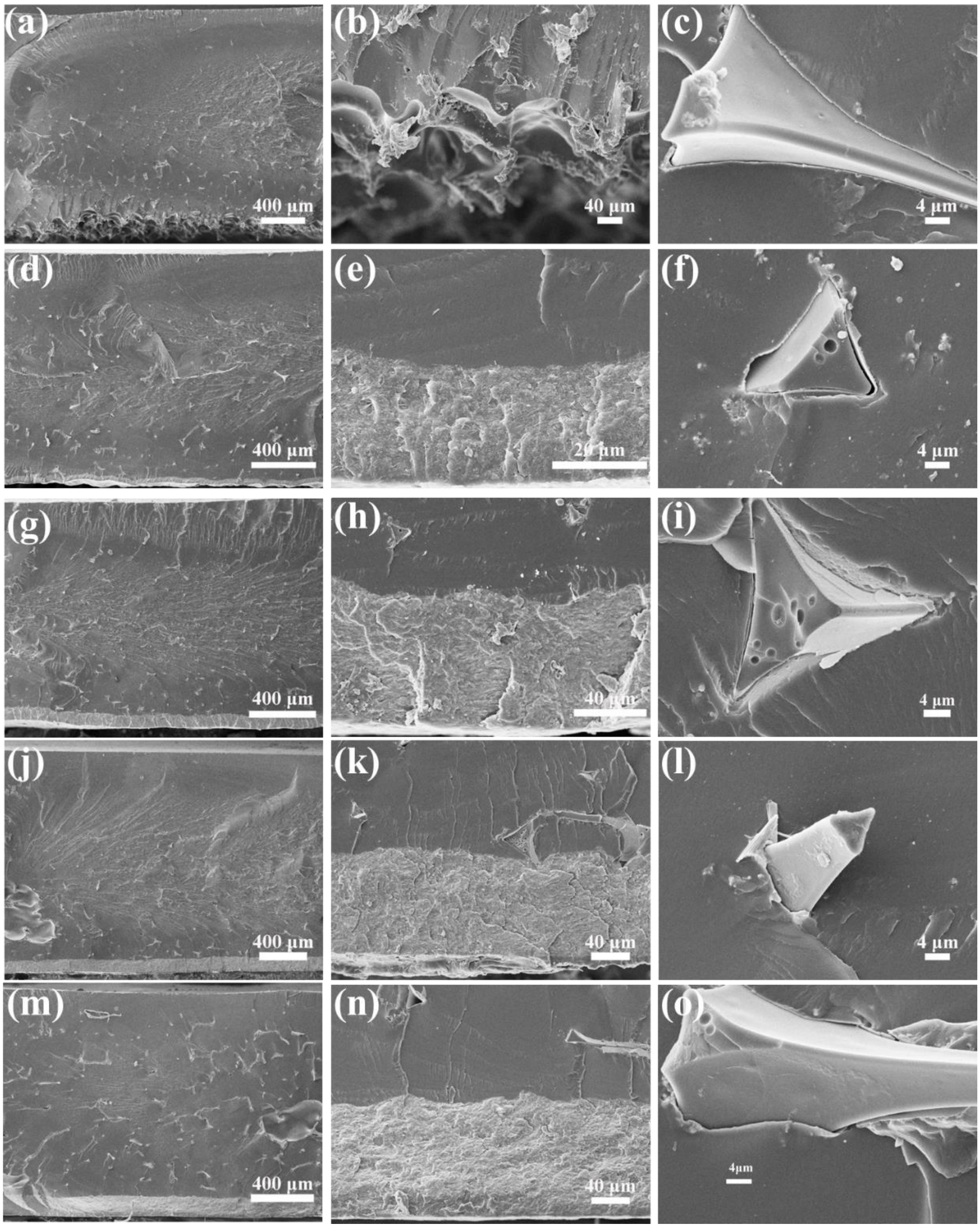

Fig. S7 SEM images of Ni@MF-3/CNT/PBAT composites with different CNT thickness. SEM images of (**a-c**) Ni@MF-3/PBAT composites, (**d-f**) Ni@MF-3/CNT-25/PBAT composites, (**g-i**) Ni@MF-3/CNT-50/PBAT composites, (**j-l**) Ni@MF-3/CNT-75/PBAT composites, and (m-o) Ni@MF-3/CNT-100/PBAT composites

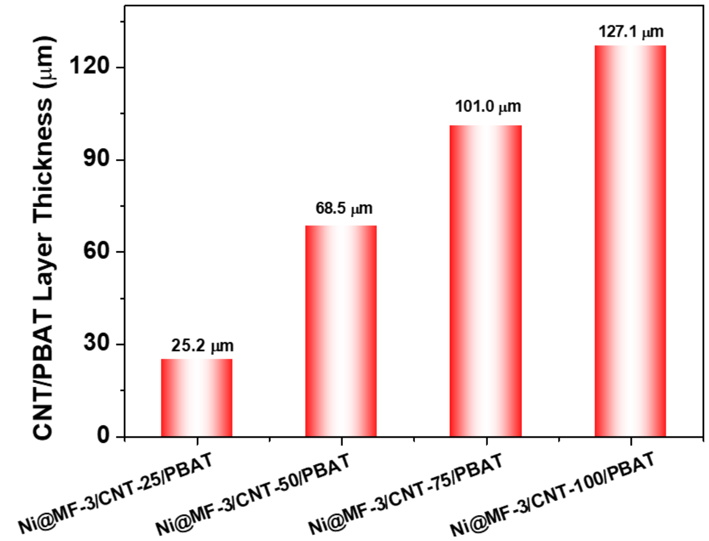

Fig. S8 The thickness of CNT/PBAT layer in Ni@MF-3/CNT/PBAT composites with different CNT paper thickness

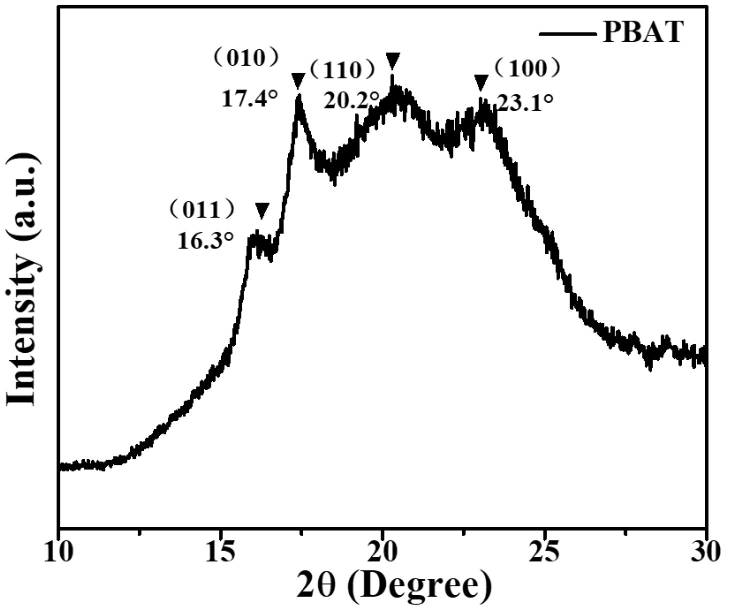

Fig. S9 The magnified XRD pattern of PBAT in 10-30°

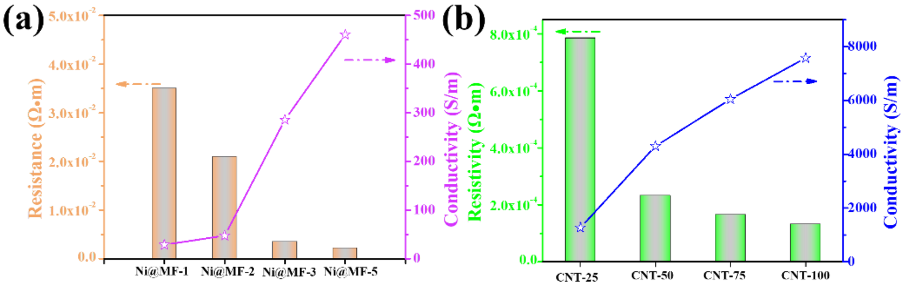

Fig. S10 Resistance and conductivity of (**a**) Ni@MF with different plating time and (**b**) CNT papers with various thickness

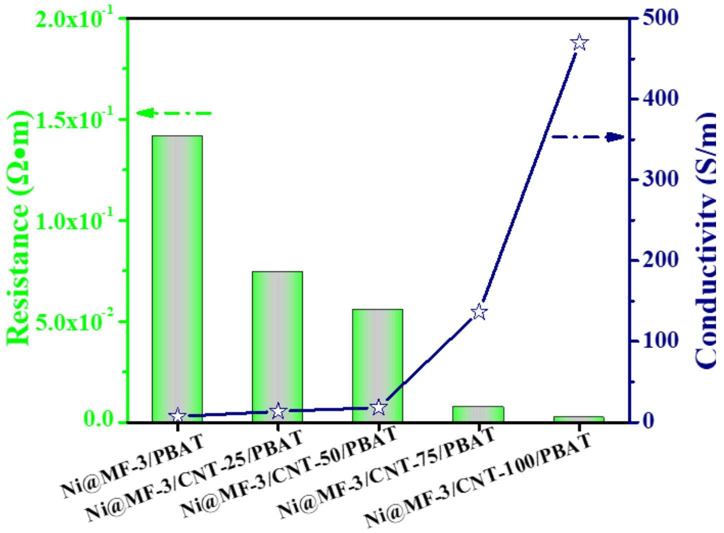

Fig. S11 Bottom surface resistance and conductivity of Ni@MF/CNT/PBAT composites

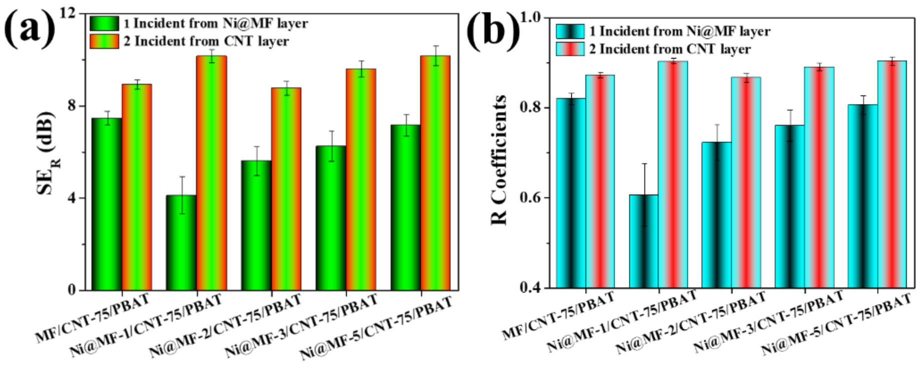

Fig. S12 The average (**a**) SE_R and (**b**) R coefficients of Ni@MF/CNT-75/PBAT composites at different incident directions

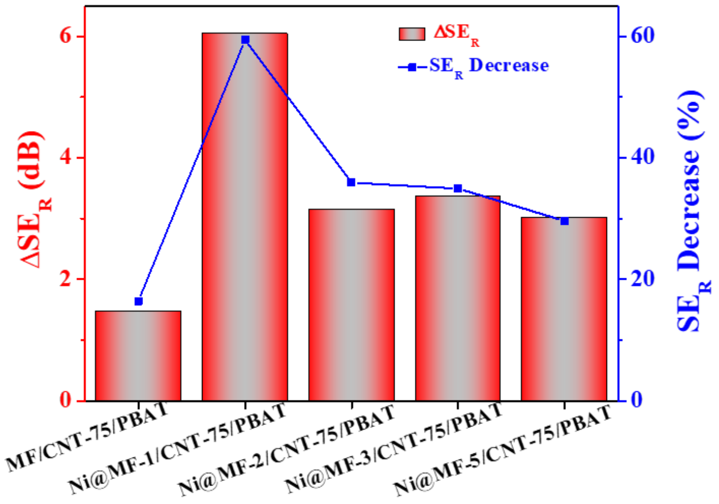

Fig. S13 ΔSE_R and SE_R enhancement of Ni@MF/CNT-75/PBAT composites at different incident directions

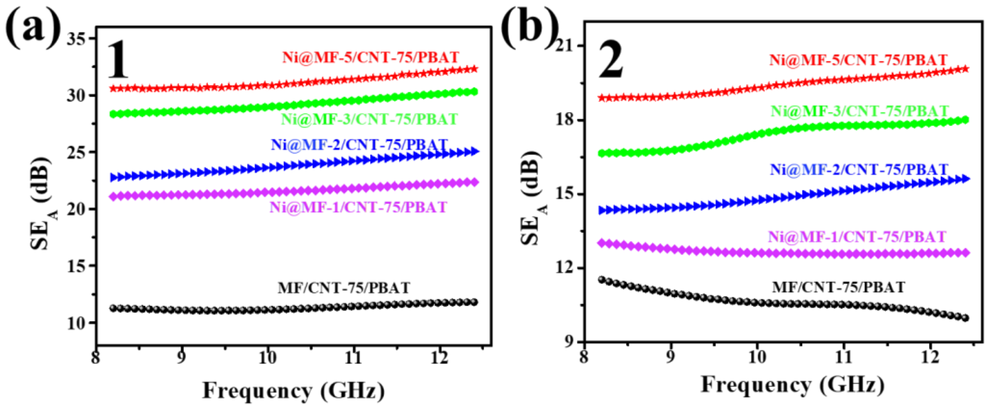

Fig. S14 EMI SE_A in X-band for Ni@MF/CNT-75/PBAT composites, when the EM wave is incident form (**a**) Ni@MF layer and (**b**) CNT layer

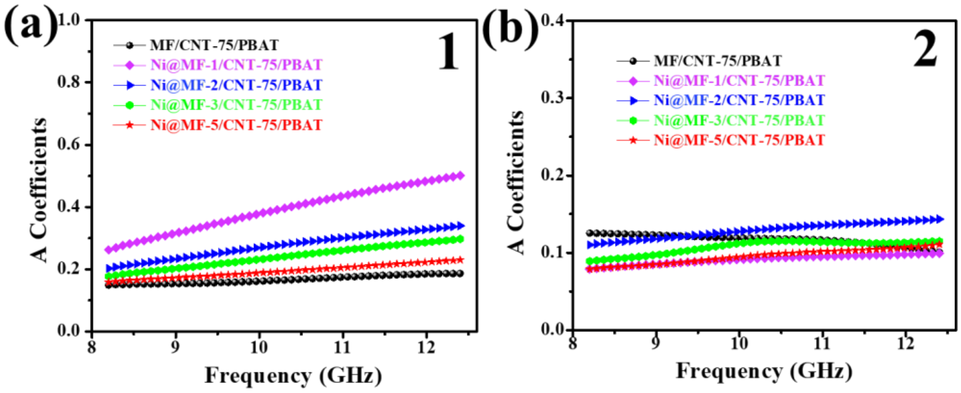

Fig. S15 A coefficients in X-band for Ni@MF/CNT-75/PBAT composites, when the EM wave is incident form (**a**) Ni@MF layer and (**b**) CNT layer

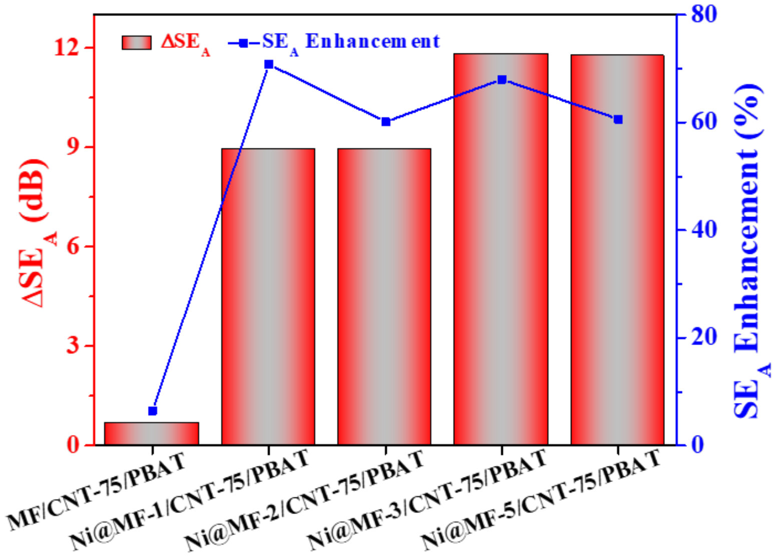

Fig. S16 ΔSE_A and SE_A enhancement of Ni@MF/CNT-75/PBAT composites at different incident directions

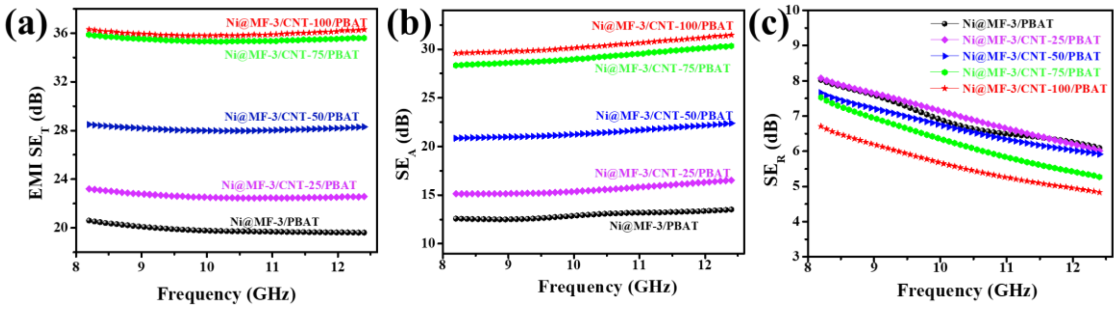

Fig. S17 (**a**) EMI SE_T, (**b**) SE_A, and (**c**) SE_R in X-band for Ni@MF-3/CNT/PBAT composites, when the EM wave is incident from Ni@MF layer

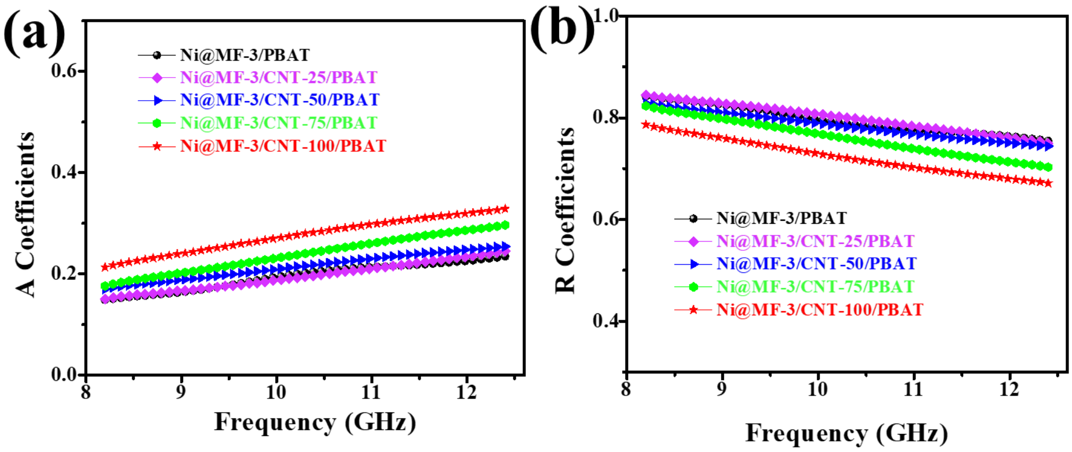

Fig. S18 (**a**) A coefficients and (**b**) R coefficients in X-band for Ni@MF-3/CNT/PBAT composites, when the EM wave is incident from Ni@MF layer

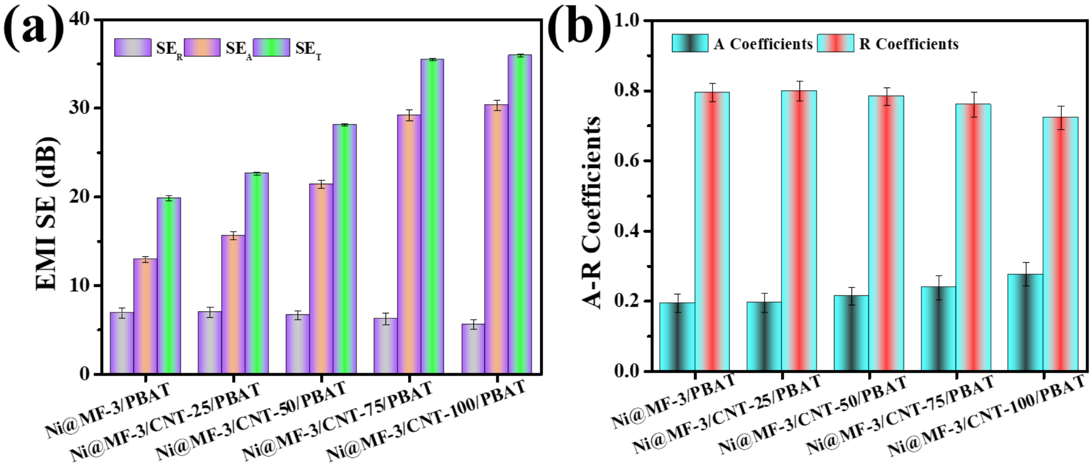

Fig. S19 (**a**) The average SE_T, SE_A, and SE_R, and (**b**) average A-R coefficient of Ni@MF-3/CNT/PBAT composites with the incident EM wave from Ni@MF layer

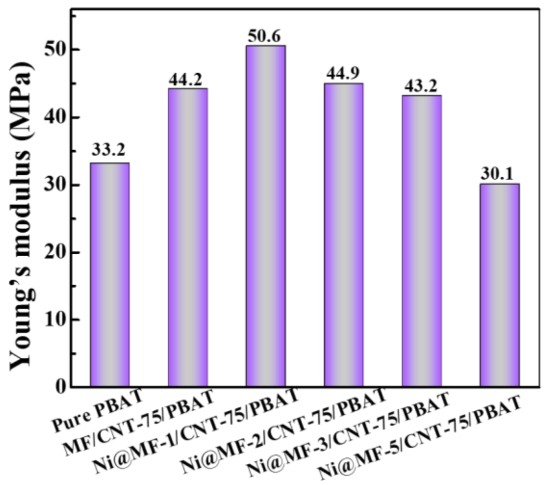

Fig. S20 The Young's modulus of pure PBAT and step-wise asymmetric Ni@MF/CNT-75/PBAT composites with different Ni plating time

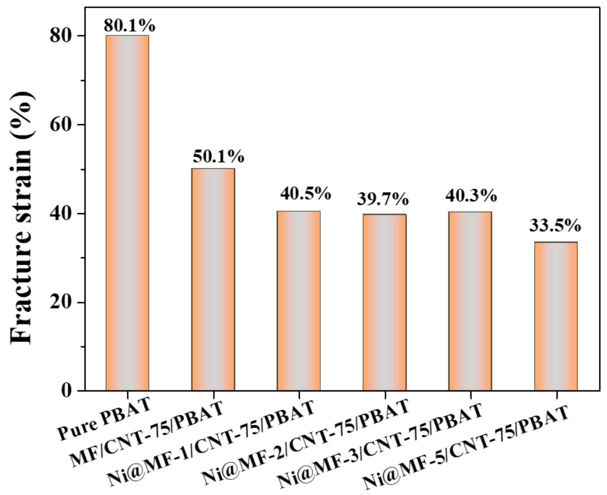

Fig. S21 The fracture strain of pure PBAT and step-wise asymmetric Ni@MF/CNT-75/PBAT composites with different Ni plating time

Table S1 Atomic percentages of pure MF and Ni@MF-5

Sample	Elemental content (at%)			
	C 1s	O 1s	N 1s	Ni 2p
**Pure MF**	66.3	14.6	19.1	-
**Ni@MF-5**	53.3	13.3	17.4	15.6

Movie S1 Practical application for directional EMI shielding
